# Kisspeptin-10 Promotes Progesterone Synthesis in Bovine Ovarian Granulosa Cells via Downregulation of microRNA-1246

**DOI:** 10.3390/genes13020298

**Published:** 2022-02-03

**Authors:** Lewei Guo, Haoran Xu, Yajun Li, Hongyu Liu, Jing Zhao, Wenfa Lu, Jun Wang

**Affiliations:** 1 Key Lab of Animal Production, Product Quality and Security, Ministry of Education, Jilin Agricultural University, Changchun 130118, China; guolewei163@163.com (L.G.); haoranxu1197@163.com (H.X.); jlndlhy0133@163.com (H.L.); jlndzjing@126.com (J.Z.); lvwenfa@jlau.edu.cn (W.L.); 2 College of Animal Science and Technology, Jilin Agricultural University, Changchun 130118, China; lyj2721933547@163.com

**Keywords:** kp-10, progesterone, bovine ovarian granulosa cells, miR-1246, StAR

## Abstract

The objective of this study was to clarify the effect of kisspeptin-10 (kp-10) on the synthesis of progesterone (P4) in bovine granulosa cells (BGCs) and its mechanisms via microRNA 1246 (miR-1246). According to the results, we found that treating with kp-10 for 24 h could increase P4 level, the mRNA expression of the steroidogenesis-related gene *steroidogenic acute regulatory protein* (StAR), free cholesterol content, and decrease miR-1246 expression in BGCs. Overexpression of miR-1246 significantly inhibited P4 synthesis, StAR mRNA expression, and free cholesterol content in BGCs, whereas underexpression of miR-1246 significantly reversed this effect in BGCs. Additionally, overexpression of miR-1246 counteracted the accelerative effect of kp-10 on P4 synthesis, StAR mRNA expression, and free cholesterol content in BGCs. Conversely, underexpression of miR-1246 enhanced the accelerative effect of kp-10 on P4 synthesis, StAR mRNA expression, and free cholesterol content in BGCs. Meanwhile, results of dual-luciferase reporter assays indicated that miR-1246 targeted the 3′UTR of StAR in BGCs. These results demonstrated that kp-10 induced P4 synthesis in BGCs by promoting free cholesterol transport via regulating expression of miR-1246/StAR.

## 1. Introduction

During follicular development in the mammalian ovaries, layers of granulosa cells (GCs) provide necessary nutrients and promote maturation for the oocytes through the special paracrine structures and junctional interactions [1,2]. Meanwhile, ovarian GCs could synthesize and secrete several steroid hormones, such as progesterone (P4) and estrogen [3]. Among them, P4 is converted from free cholesterol through diverse rate-limiting enzymes, including steroid-derived acute regulatory enzyme (StAR), 3-β-hydroxysteroid dehydrogenase, and P450 side chain lyase [4]. Furthermore, in female animals, P4 is involved in the regulation of physiological processes such as menstrual cycle, pregnancy, and lactation [5,6,7]. In view of the central role of GC in the ovary, it is of great significance to clarify the mechanism of P4 synthesis in GCs.

Kisspeptin is a polypeptide encoded by the *KISS-1* gene [8]. Kisspeptin is cleaved to C-terminal peptide into four biologically active peptides including kisspeptin-54, kisspeptin-14, kisspeptin-13, and kisspeptin-10 (kp-10) [9], which have similar biological activities and functions. Kisspeptin could stimulate the synthesis and secretion of gonadotropin-releasing hormone (GnRH) by acting directly on the G protein-coupled receptor 54 (GPR54) receptor of GnRH neurons [10]. Meanwhile, kisspeptin and GnRH may act on the reproductive axis to regulate reproductive activities [11,12]. Several studies have supported kisspeptin expression in the GCs of ovaries of different animals [13,14,15]. The expression of kisspeptin and *KISS1R* in buffalo ovary is affected by the follicles, and the expression is higher in the GCs of the follicles with high P4 concentration [16], indicating the potential relationship between kisspeptin and P4 synthesis. Previous studies suggest that kisspeptin could stimulate P4 synthesis in porcine, chicken, rat, and bat ovarian GCs [17,18,19,20]. However, the effect and mechanism of kp-10 on P4 synthesis in bovine ovarian GCs (BGCs) is still unclear.

MicroRNAs (miRNAs) are small non-coding RNAs that negatively regulate gene translation by binding complementary sequences within 3′-untranslated regions (3′-UTRs) [21]. miRNAs also exert vital functions in the regulation of follicular growth and development. For example, miR-20a, miR-224, miR-383, and miR-378 regulate steroid hormone synthesis in GCs [22,23,24,25]. miR-1246 has been reported to be an oncogenic miRNA that is involved in the migration and invasion of colon cancer, acute myeloid leukemia, esophageal squamous cell carcinoma, breast cancer, and other cancer cells [26,27,28,29]. In a preliminary study, we found that kp-10 could decrease miR-1246 expression in BGCs (data unpublished). A recent study found that P4 levels of buffalo GCs increased under heat stress, accompanied by a decrease in the expression of miR-1246, suggesting that miR-1246 may be associated with P4 synthesis [30]. Bioinformatics analyses revealed that StAR is a potential target gene of miR-1246. StAR controls the transport of free cholesterol (a precursor of steroid hormones) from the mitochondrial outer membrane to the mitochondrial inner membrane, and it is the rate-limiting entity of P4 synthesis [31]. Therefore, we hypothesized that kp-10 affects P4 synthesis through miR-1246 targeting StAR and regulates free cholesterol transport in BGCs. Our founding of this work will provide initial insights for further research and help us to understand the molecular mechanisms of P4 synthesis from GCs.

## 2. Materials and Methods

### 2.1. Primary BGC Culture 

Fresh bovine ovaries were collected in the local slaughterhouse (Changchun, China). The fresh ovaries were brought to the laboratory within 3 h. BGCs were aspirated from ovarian follicles (diameters 3–6 mm) with 5 mL sterile syringes. Cell suspensions were washed with sterile PBS buffer supplemented for several times. Final inoculated 1–1.2 × 10^6^ BGCs into the 60 mm cell culture plates, and cultured in an incubator with 5% CO_2_ at 37 °C. Meanwhile, BGCs were cultured in DMEM/F-12 (Gibco, Thermo Scientific, New Castle, DE, USA) containing 10% FBS (Gibco). All animal experiments were approved by the Animal Ethics Committee of the Jilin Agricultural University.

### 2.2. Bovine Granulosa Cells (BGCs) Treatment

BCGs were divided into following groups: CTR group (blank control group), kp-10 group (treated with 100 nM kp-10 purchased from ChinaPeptides Co., Ltd., Shanghai, China, for 24 h), mimics negative control (NC) group (transfected with mimics NC), miR-1246 mimic group (transfected with miR-1246 mimics), inhibitor NC group (transfected with inhibitor NC), miR-1246 inhibitor group (transfected with miR-1246 inhibitor), mimics NC + kp-10 group (treated with mimics NC + 100 nM kp-10), miR-1246 mimic + kp-10 group (treated with miR-1246 mimics + 100 nM kp-10), inhibitor NC + kp-10 group (treated with inhibitor NC + 100 nM kp-10), and miR-1246 inhibitor + kp-10 group (transfected with miR-1246 inhibitor + 100 nM kp-10).

The bovine miR-1246 mimics and inhibitor were synthesized by Sangon Biotech Co., Ltd. (Shanghai, China, Table 1). Based on the manufacturer’s standards, BGCs were transfected by Lipofectamine™ 2000 (Invitrogen, Carlsbad, CA, USA). In brief, 500 pmol miR-1246 mimic (mimics NC) or miR-1246 inhibitor (inhibitor NC) was added to serum-free media and then mixed with 20 μL of Lipofectamine™ 2000 per well, stand for 20 min at room temperature. The mixture was added to each culture dish at 37 °C in 5% CO_2_. After 6 h, the cell culture medium was replaced with fresh medium and the cells were cultured for 42 h. Among them, after transfection for 24 h, the mimics NC + kp-10 group, miR-1246 mimic + kp-10 group, inhibitor NC + kp-10 group, and miR-1246 inhibitor + kp-10 group were treated with 100 nM kp-10 for 24 h.

### 2.3. Progesterone (P4) Enzyme-Linked Immunosorbent Assay (ELISA)

BGC supernatants were collected and the levels of bovine P4 synthesis were quantified using an ELISA kit (MM-50918O2, Meimian, Wuhan, China). The assay was performed based on the manufacturer’s standards. Briefly, the samples and reagents of the ELISA kit were mixed, washed, and incubated at 37 °C. The absorbance at 450 nm was set using an Epoch microplate reader (Biotek, Winooski, VT, USA), and blank correction was performed for all obtained data.

### 2.4. Reverse-Transcription Quantitative PCR (qRT-PCR)

Total RNA was extracted from BGCs using the TRIzol method (TaKara, Shiga, Japan) according to the manufacturer’s standards, and all corresponding steps were carried out on ice. Reverse transcription to produce cDNA was performed using the PrimeScript RT reagent kit with gDNA Eraser (TaKara). Expression of StAR and miR-1246 was detected using specific primers (Table 2). The PCR conditions were as follows: 95 °C 30 s; 40 × (95 °C 5 s, 60 °C/64 °C 34 s); 95 °C 15 s; 60 °C 60 s; 95 °C 15 s. Taking β-actin as reference, three technical repetitions for each treatment were used. The 2^−ΔΔCt^ method was used to analyze the data.

### 2.5. Filipin Staining

To determine the BGCs’ free cholesterol accumulation, the BGCs were grown on glass bottom plates in the same culture conditions as otherwise. The cells were washed with PBS and fixed for 30 min with 4% paraformaldehyde. Then, cells were treated with 0.3% Triton-X 100 for 5 min. Subsequently, the cells were stained with filipin working solution (50 μg/mL) (Sigma-Aldrich, St. Louis, MO, USA) for 1 h at 37 °C, and stained with Hoechst33258 for 10 min at 37 °C. After several washes in PBS, the cells were mounted on a glass slide. The images were captured using a full-functional cell imaging detector (BioTek, Winooski, VT, USA), at least five random microscope fields were selected in each experimental group.

### 2.6. Cholesterol Assay

Free cholesterol content was measured by Free Cholesterol Assay Kits (Applygen Technologies, Beijing, China). Cellular protein content was determined based on a BCA assay by using a Byotime (Shanghai, China) kit. Each experiment was performed in triplicate. 

### 2.7. Dual-Luciferase Reporter Assays

The 3′-UTR of StAR with the wild type (WT) and mutant (MUT) binding sites of miR-1246 were synthesized by Sangon Biotech Co., Ltd. (Shanghai, China), and the sequence information was shown in Table S1. Sequences were cloned into a psiCHECK-2 vector (Promega, Madison, WI, USA) between the XhoI and SmaI restriction sites and expression was regulated using a SV40 promoter. HEK293T cells were divided into following groups: miRNA mimics + StAR-WT group (mimics NC co-transfected with psiCHECK-StAR-WT), miR-1246 mimics + StAR-WT group (miR-1246 mimics co-transfected with psiCHECK-StAR-WT), miRNA mimics + StAR-MUT group (mimics NC cotransfected with psiCHECK-StAR-MUT), and miR-1246 mimics + StAR-MUT group (miR-1246 mimics cotransfected with psiCHECK-StAR-MUT). The concentration of psiCHECK-StAR-WT or psiCHECK-StAR-MUT reporter plasmids were 50 nM and cells were transfected for 48 h. Collected the cells and analyzed Firefly and Renilla luciferase activities using the dual-luciferase reporter system (Promega, Madison, WI, USA). Luciferase activity was standardized to the respective Renilla luciferase activity.

### 2.8. Statistical Analyses

Data were presented as the mean ± standard deviation of the mean. Statistical analysesused Student’s *t*-test (SPSS version 23.0, Chicago, IL, USA). Then the GraphPad Prism Software (GraphPad Software, San Diego, CA, USA) was employed to draw figures. Differences with *p* < 0.05 were considered statistically significant.

## 3. Results

### 3.1. Effect of kp-10 on Expression of miR-1246 and P4 Synthesis in BGCs

**Figure 1:**
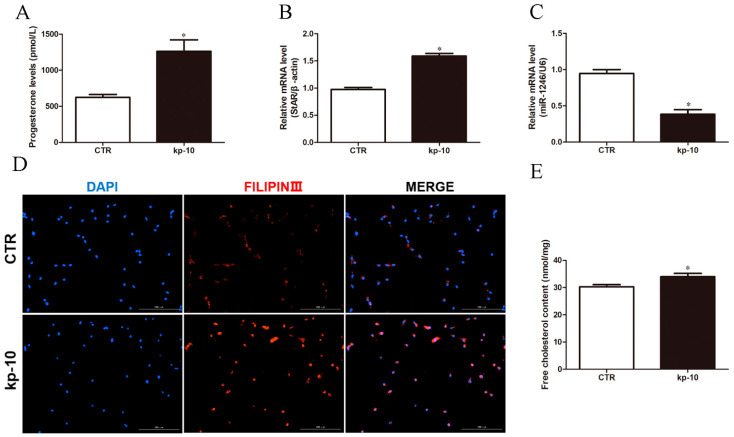
kp-10 promotes P4 synthesis and reduces miR-1246 expression in BGCs. (**A**) The level of P4 in BGCs. (**B**,**C**) The relative mRNA expression of StAR and miR-1246 in BGCs. (**D**) Representative filipin staining of BGCs. (**E**) Relative content of free cholesterol in BGCs. All data were presented as the mean ± SD (*n* = 3), * *p* < 0.05.

To explore the functional roles of kp-10 in BGCs, we treated the cells with or without kp-10 for 24 h. As shown in Figure 1A, compared to the CTR group, BGCs with kp-10 treated significantly increased level of P4 (*p* < 0.05). Meanwhile, qRT-PCR analysis reflected BGCs with kp-10 treated significantly increased steroidogenesis-related gene *StAR* mRNA expression (*p* < 0.05; Figure 1B), and significantly decreased *miR-1246* mRNA expression (*p* < 0.05; Figure 1C). Moreover, filipin staining indicated that kp-10 treatment evidently developed excess free cholesterol (increased red dots) in BGCs (Figure 1D). Notably, the free cholesterol content in each group were detected by Free Cholesterol Assay Kits. We found the free cholesterol content of kp-10 treatment was significantly increased (*p* < 0.05; Figure 1E). These results indicated that kp-10 could promote P4 synthesis by increasing StAR mRNA expression and free cholesterol content and reducing miR-1246 expression in BGCs.

### 3.2. Effects of miR-1246 on P4 Synthesis in BGCs

**Figure 2:**
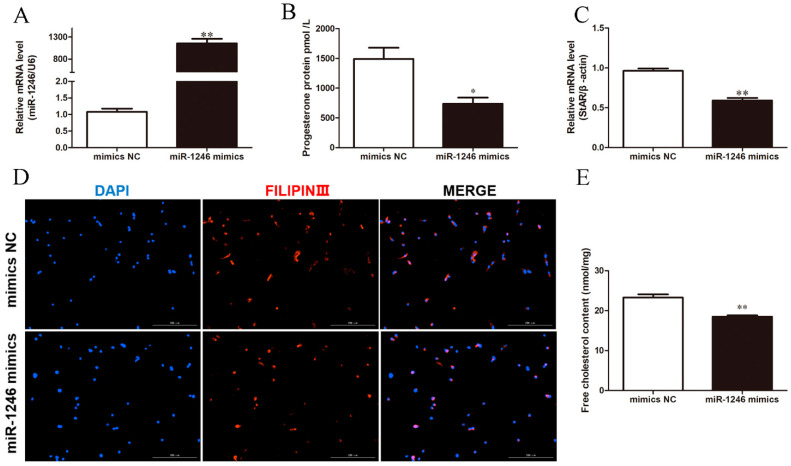
Overexpression of miR-1246 reduces P4 synthesis in BGCs. (**A**) The level of P4 in BGCs. (**B**,**C**) The relative mRNA expression of StAR and miR-1246 in BGCs. (**D**) Representative filipin staining of BGCs. (**E**) Relative content of free cholesterol in BGCs. Mimics NC, negative control of miR-1246 mimics; miR-1246 mimics, overexpression of miR-1246. All data were presented as the mean ± SD (*n* = 3), * *p* < 0.05, ** *p* < 0.01.

**Figure 3:**
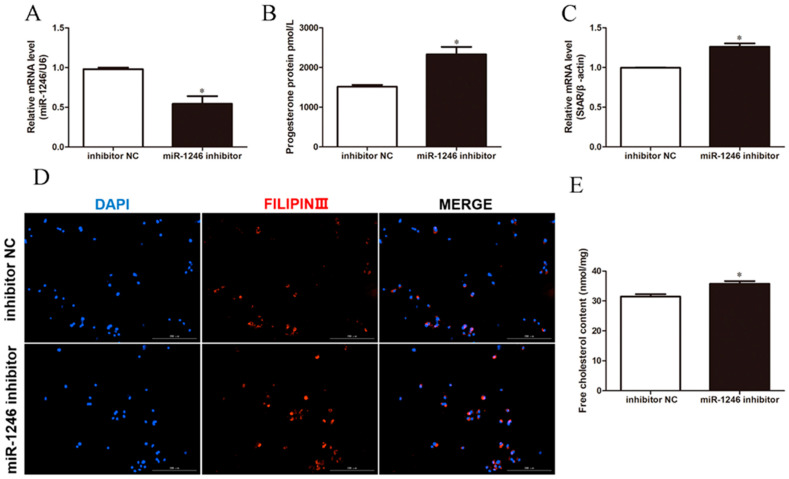
Underexpression of miR-1246 promotes P4 synthesis in BGCs. (**A**) The level of P4 in BGCs. (**B**,**C**) Relative mRNA expression of StAR and miR-1246 in BGCs. (**D**) Representative filipin staining of BGCs. (**E**) Relative content of free cholesterol in BGCs. Inhibitor NC, negative control of miR-1246 inhibitor; miR-1246 inhibitor, underexpression of miR-1246. All data were presented as the mean ± SD (*n* = 3), * *p* < 0.05.

To understand the effects of miR-1246 on P4 synthesis in BGCs, we transfected BGCs with miR-1246 mimics or miR-1246 inhibitor for 48 h. As expected, transfection with miR-1246 mimics significantly increased miR-1246 expression (*p* < 0.05; Figure 2A). Overexpression of miR-1246 decreased the levels of P4 and the mRNA expression of StAR (*p* < 0.05; Figure 2B,C). Filipin staining and cholesterol assay revealed that transfection of miR-1246 mimics decreased the concentration of free cholesterol (Figure 2D,E). In contrast, transfection with the miR-1246 inhibitor decreased miR-1246 expression (*p* < 0.05; Figure 3A) and increased P4 levels and StAR mRNA expression (*p* < 0.05; Figure 3B,C). Compared with the inhibitor NC group, miR-1246 inhibitor also increased the concentration of free cholesterol (Figure 3D,E). These results demonstrated that miR-1246 could reduce P4 synthesis by decreasing the mRNA expression of StAR and the content of free cholesterol in BGCs.

### 3.3. Kp-10 Affects P4 Synthesis of BGCs by Regulating miR-1246 Expression

**Figure 4:**
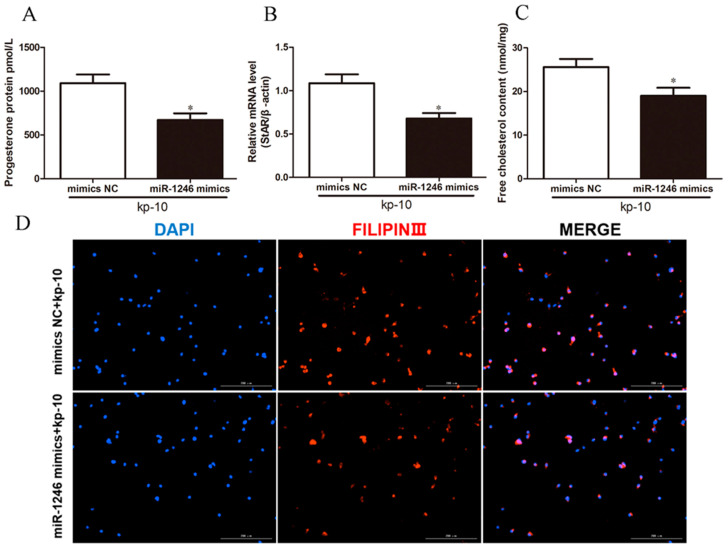
Overexpression of miR-1246 attenuated the accelerative effect of kp-10 on P4 synthesis of BGCs. (**A**) The level of P4 in BGCs. (**B**) Relative StAR mRNA expression in BGCs. (**C**) Representative filipin staining of BGCs. (**D**) Relative free cholesterol content in BGCs. Mimics NC, negative control of miR-1246 mimics; miR-1246 mimics, overexpression of miR-1246. All data were presented as the mean ± SD (*n* = 3), * *p* < 0.05.

**Figure 5:**
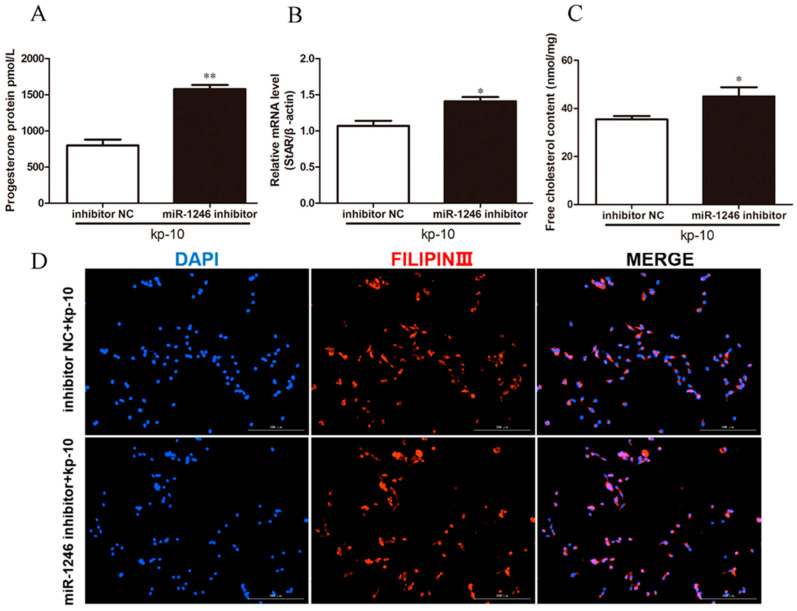
miR-1246 inhibitor enhanced the effect of kp-10 on P4 synthesis of BGCs. (**A**) The level of P4 in BGCs. (**B**) Relative StAR mRNA expression in BGCs. (**C**) Representative filipin staining of BGCs. (**D**) Relative free cholesterol content in BGCs. Inhibitor NC, negative control of miR-1246 inhibitor; miR-1246 inhibitor, underexpression of miR-1246. All data were presented as the mean ± SD (*n* = 3), * *p* < 0.05, ** *p* < 0.01.

To investigate whether miR-1246 mediates the effects of kp-10 on P4 synthesis in BGCs, miR-1246 mimics/mimics NC or miR-1246 inhibitor/inhibitor NC were transfected for 24 h and then treated with kp-10 for 24 h. We found that overexpression of miR-1246 attenuated the accelerative effect of kp-10 on P4 synthesis of BGCs (*p* < 0.05; Figure 4A). Furthermore, miR-1246 mimics transfects of kp-10-treated BGCs decreased the mRNA expression of StAR (*p* < 0.05; Figure 4B), while decreasing the content of free cholesterol (Figure 4C,D). Also, the miR-1246 inhibitor enhanced the effect of kp-10 on P4 synthesis of BGCs (*p* < 0.05; Figure 5A). Moreover, miR-1246 inhibitor transfects of kp-10-treated BGCs increased the mRNA expression of StAR (*p* < 0.05; Figure 5B) and the content of free cholesterol (Figure 5C,D). Overall, these results suggest that kp-10 promotes P4 synthesis in BGCs by downregulating miR-1246 expression.

### 3.4. miR-1246 Targets StAR Expression in BGCs

**Figure 6:**
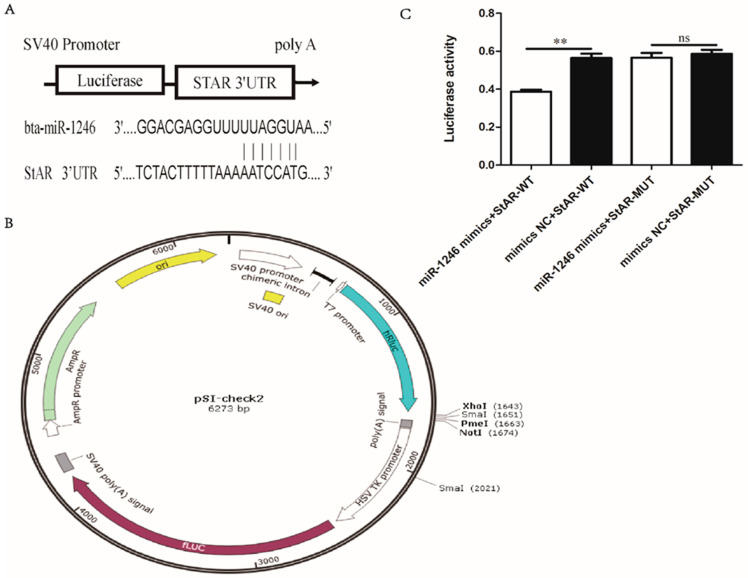
StAR was a target of miR-1246 in BGCs. (**A**) Predicted miR-1246 binding site in the StAR 3′UTR. (**B**) The luciferase reporter vector fused with StAR 3′UTR-WT or its MUT was constructed. (**C**) Dual-luciferase assay measuring the luciferase activity in different treatment groups of BGCs. Mimics NC, negative control of miR-1246 mimics; miR-1246 mimics, overexpression of miR-1246; WT, wild type; MUT, mutant. All data were presented as the mean ± SD (*n* = 3), ** *p* < 0.01, ns means no significance.

To further study the binding targets of miR-1246, we predicted the binding sites between the bta-miR-1246 and target genes and discovered that bta-miR-1246 and bovine *StAR* gene have a binding site (Figure 6A). To validate the miR-1246 target gene, the 3′UTR WT or its MUT was cloned into a reporter vector (Figure 6B), and a standard luciferase reporter assay was conducted. Dual-luciferase activity assay result demonstrated that the ‘miR-1246 mimics + StAR-WT’ group was more significantly suppressing the luciferase activity than the ‘mimic NC + StAR-WT’ group; whereas the luciferase activity did not change between the ‘mimic NC + StAR-MUT’ group and ‘miR-1246 mimics + StAR-MUT’ groups (Figure 6C). Considering the negative regulation of miR-1246 on StAR, we found that StAR was a targeted miR-1246. Taken together, these results signified that kp-10 promotes P4 synthesis through miR-1246 targeting StAR and regulates free cholesterol transport in BGCs.

## 4. Discussion

GCs are the main somatic cells of ovarian follicles, which are important in the recruitment of primordial follicles, follicular development, atresia, ovulation, and luteinization [32,33]. Furthermore, GCs could secrete the necessary hormones to support the maintenance, growth, and quality of oocytes, such as P4 and estradiol [34,35]. In this study, we demonstrated that kp-10 promoted P4 synthesis by regulating the content of free cholesterol in BGCs. We also found that miR-1246 mediated kp-10-induced P4 synthesis by targeting StAR in BGCs.

Kp-10 not only regulates mammalian reproductive activity through the hypothalamus but also directly acts on the ovary and regulates follicular development and ovulation [36,37]. Although, previous studies have shown that kisspeptin treatment stimulated P4 production in cultured porcine, chicken, rat, and bat ovarian GCs, the mechanism of action of kisspeptin is unclear. In this study, we used miRNA as an entry point and found that kp-10 reduced the expression of miR-1246 while promoting P4 synthesis and steroidogenesis-related gene *StAR* expression in BGCs. This result implies that, although there had important species differences among porcine, chicken, rat, bat, and bovine, kp-10 had similar functions in GCs of different species, and kp-10 might regulate P4 synthesis through miR-1246.

Recently, it has been widely accepted that miRNAs were involved in the regulation of P4 synthesis in GCs [38]. A recent study showed that kp-10 stimulated Senegalese soles’ gonadotropin and testosterone synthesis and induced changes in its plasma miRNAs [39]. Similarly, we found that kp-10 decreased miR-1246 expression in BGCs. miR-1246 is involved in the regulation of ovarian functioning, as observed when human amniotic epithelial cell-derived exosomes were used to restore ovarian functions by transferring miR-1246 to induce apoptosis [40]. miR-1246 was also identified as a promising diagnostic biomarker of high-grade serous ovarian carcinoma [41]. Under heat stress, the P4 levels were increased in buffalo GCs, accompanied by a decrease in the expression of miR-1246 [30], further suggesting that miR-1246 may be associated with P4 synthesis. Based on these observations, we found that overexpression of miR-1246 could inhibit BGCs’ P4 synthesis, whereas underexpression of miR-1246 reversed this effect in BGCs. In this study, our findings confirmed that miR-1246 decreased P4 synthesis and StAR mRNA expression in BGCs. To our knowledge, this is the first direct evidence that miR-1246 is involved in P4 synthesis in BGCs.

miRNAs could mediate gene expression by targeting the specific site of the 3′UTR of genes in mammals. In germ cells, miR-301a-5p was involved in the transforming growth factor-beta (TGF-β) signaling pathway by targeting TGF-β2 to promote cell development [42]. In porcine ovarian GCs, miR-31 targets 17-beta-hydroxysteroid dehydrogenase 14 (HSD17B14) and follicle-stimulating hormone receptor, and miR-20b targets HSD17B14 to affect cell apoptosis and the P4 concentration [43]. Similarly, we found miR-1246 affects the P4 synthesis in BGCs by targeting StAR.

Free cholesterol is the precursor of P4 synthesis, which is largely provided by the hydrolysis of cholesteryl esters (CE) stored in lipid droplets [44]. StAR could control the transport of free cholesterol from the mitochondrial outer membrane to the mitochondrial inner membrane for P4 synthesis, and it is the rate-limiting entity of P4 synthesis [31]. In MA-10 Leydig cells, the hydrolysis of CE to free cholesterol and promote the expression of StAR could promote P4 synthesis [45]. Similarly, we found that the change of *StAR* gene expression is consistent with the change trend of free cholesterol content in BGCs. There are also some limitations in the present study. Since we only studied the effect of kp-10 on P4 synthesis in BGCs in vitro but did not design an experiment in vivo, we could not confirm whether kp-10 affects the physiological function of bovine ovaries. This part will be the focus of our next project.

## 5. Conclusions

Our results demonstrated that kp-10 promotes P4 synthesis through miR-1246 targeting StAR and regulates free cholesterol transport in BGCs. They also revealed the importance of kp-10 in the regulation of P4 synthesis in BGCs, which may help improve our understanding of the mechanisms underlying GC P4 synthesis and may facilitate the development of novel strategies to counteract bovine ovarian follicle atresia.

## Figures and Tables

**Table 1:** Sequences of miR-10b and mimics NC.

Name	Sequence(5′–3′)
bta-miR-1246 mimics	Sense: AAUGGAUUUUUGGAGCAGG
	Antisense: UGCUCCAAAAAUCCAUUUU
bta-mimics NC	Sense: UUGUACUACACAAAAGUACUG
	Antisense: GUACUUUUGUGUAGUACAAUU
bta-miR-1246 inhibitor	CCUGCUCCAAAAAUCCAUU
bta-inhibitor NC	CAGUACUUUUGUGUAGUACAA

**Table 2:** Sequences of miR-10b and mimics NC.

Primer Name	Primer Sequence(5′–3′)	Annealing Temperatures/°C
miR-1246 RT	GTCGTATCCAGTGCAGGGTCCGAGGTATTCGCACTGGATACGACCCTGCT	--
miR-1246	F: GCGCGAATGGATTTTTGG	64
	R: AGTGCAGGGTCCGAGGTATT	
StAR	F: AAGACCCTCTCTACAGCGACCAAG	60
	R: CTCTCCTTCTTCCAGCCCTCCTG	
U6	F: GCTTCGGCAGCACATATACTAAAAT	64
	R: CGCTTCACGAATTTGCGTGTCAT	
β-actin	F: TTGATCTTCATTGTGCTGGGTG	60
	R: CTTCCTGGGCATGGAATCCT	

## Data Availability

The raw data supporting the conclusions of this article will be made available by the authors, without undue reservation.

## Supplementary Materials

The following are available online at https://www.mdpi.com/article/10.3390/genes13020298/s1, Table S1: StAR 3′UTR sequence.
