# Supplementary material for: Kisspeptin-10 Promotes Progesterone Synthesis in Bovine Ovarian Granulosa Cells via Downregulation of microRNA-1246
Source: Genes (Basel). 2022 Feb 3;13(2):298. doi: 10.3390/genes13020298 (PMC8871966; doi:10.3390/genes13020298)
Supplement: Supplementary file 1 [file genes-13-00298-s001.zip › genes-1568036-SI.pdf]

Table S1. StAR 3’UTR sequence

	3’UTR sequence
StAR-WT	CTCGAGACAGCTGCACCAAAGAGTAAGCAACTCCCACAGCAGACGGCTTCT<br>AGAACTCTAGTTCAAGTGACTTACGGAAAAAATACAGAACTGTTATACTGA<br>TTCCCGTACTTCTTCCATGACAGGAGTCAGAATAAAGAATTGTAACTAACAT<br>AAAAACTTTCAGTTAAGTCTGTACCCGATTTAAAATTCTACTTTTTAAAAAT<br>CCATGCTAATAAATGGCAAGCTCATACTAAAGGAGCCGTGGATAAAGATTT<br>TAATTAAACTAAATTTCTTACTTCATTCAAAGGAAAAACTCCAGGGGACTTA<br>AGAATTTCAATTATGTAGGATGTTACTGGAATCTTTCATAAAAATTTAATTT<br>GGAAAATACGCACAAGACTAAATCAGTTCTTACAAGAACTCTAGCGGCCGC
StAR-MUT	CTCGAGACAGCTGCACCAAAGAGTAAGCAACTCCCACAGCAGACGGCTTCT<br>AGAACTCTAGTTCAAGTGACTTACGGAAAAAATACAGAACTGTTATACTGA<br>TTCCCGTACTTCTTCCATGACAGGAGTCAGAATAAAGAATTGTAACTAACAT<br>AAAAACTTTCAGTTAAGTCTGTACCCGATTTAAAATTCTACTTTTTAAACAG<br>CAAGGCTAATAAATGGCAAGCTCATACTAAAGGAGCCGTGGATAAAGATTT<br>TAATTAAACTAAATTTCTTACTTCATTCAAAGGAAAAACTCCAGGGGACTTA<br>AGAATTTCAATTATGTAGGATGTTACTGGAATCTTTCATAAAAATTTAATTT<br>GGAAAATACGCACAAGACTAAATCAGTTCTTACAAGAACTCTAGCGGCCGC

Note: The cloned target sequence is shown in grey, and the predicted binding site to miR-1246 is shown in green.
